# Second-to-Fourth Digit Ratio Has a Non-Monotonic Impact on Altruism

**DOI:** 10.1371/journal.pone.0060419

**Published:** 2013-04-10

**Authors:** Pablo Brañas-Garza, Jaromír Kovářík, Levent Neyse

**Affiliations:** 1 Business School, Middlesex University London, London, United Kingdom; 2 Dpto. Fundamentos Análisis Económico I & BRiDGE, University of the Basque Country, Bilbao, Spain; 3 GLOBE: Department of Economics, Universidad de Granada, Granada, Spain; Harvard University, United States of America

## Abstract

Gene-culture co-evolution emphasizes the joint role of culture and genes for the emergence of altruistic and cooperative behaviors and behavioral genetics provides estimates of their relative importance. However, these approaches cannot assess which biological traits determine altruism or how. We analyze the association between altruism in adults and the exposure to prenatal sex hormones, using the second-to-fourth digit ratio. We find an inverted U-shaped relation for left and right hands, which is very consistent for men and less systematic for women. Subjects with both high and low digit ratios give less than individuals with intermediate digit ratios. We repeat the exercise with the same subjects seven months later and find a similar association, even though subjects' behavior differs the second time they play the game. We then construct proxies of the median digit ratio in the population (using more than 1000 different subjects), show that subjects' altruism decreases with the distance of their ratio to these proxies. These results provide direct evidence that prenatal events contribute to the variation of altruistic behavior and that the exposure to fetal hormones is one of the relevant biological factors. In addition, the findings suggest that there might be an optimal level of exposure to these hormones from social perspective.

## Introduction

Human societies are built on cooperation and social norms [1]–[3]. It is thus important to understand the origins and determinants of prosocial behavior in humans. Gene-culture co-evolution stresses the joint role of culture and genes for the emergence of altruistic and cooperative traits [4], [5] and behavioral genetics has recently provided estimates of their relative importance, by comparing monozygotic twins who share 100% of their genes with dizygotic twins who share 50% of genes on average [6]–[8]. The limitation of these approaches is that they are unable to disentangle which particular biological traits determine individual differences in prosociality and how they are related [9]. The prominent or at-risk individuals can be those for whom the traits have low or large values, or a non-monotonic association may exist. Non-monotonicity may be particularly important in case of biological traits, since they are shaped by evolutionary forces toward “optimal” values [10]–[12] and deviations from these values in any direction might matter. Such an argument is supported by Nye et al. [13] who find systematic non-monotonic associations between digit ratio and several measures of academic performance.

To determine which traits matter and how is crucial to further understanding of the origins and individual variation of human prosociality, to the interpretation of correlations between prosocial behavior and neural activities in the brain, and to any policy targeting prosociality, cooperation and participation in the commons.

We analyze whether altruism [14]–[16] may be shaped by exposure to prenatal sex hormones. The exposure to male and female sex hormones in uterus around the end of the first trimester of pregnancy has large organizing effects on human brain development [17]. Since the neuroeconomic evidence detects that the activity in specific brain areas such as the striatum or insula correlates with altruistic behavior [18]–[20], different exposure to prenatal hormones, especially testosterone or oestrogen, may affect these areas. We thus suspect that exposure to fetal hormones may shed light on why some people are more or less selfish.

We use giving in the Dictator Game (DG) as a measure of altruism and both left- and right-hand second-to-fourth digit ratio (2D:4D) as a biomarker of exposure to fetal sex hormones. DG is a situation, in which one subject, Dictator, decides the division of a fixed amount of money (5€ in our experiment) between herself and another anonymous person, Receiver. The Dictator can hold the whole amount for herself or she can share any part of the money with the Receiver. Since giving is costly for the Dictator and the Receiver cannot affect the proposed distribution, Dictators' giving is interpreted as an act of altruism and the amount given to the Receiver serves as a measure of Dictators' altruism. Since Dictators do not know the identity of Receivers (and viceversa), altruism is therefore interpreted here as the willingness to share voluntarily with unknown individuals at subjects' cost in a reciprocity-free environment.

2D:4D is calculated as the ratio between the lengths of index and ring fingers and it has been documented that 2D:4D is inversely related to high exposure to testosterone and low exposure to oestrogen while in uterus [21]–[25].

Due to hormone exposure, men have lower 2D:4D's than women [25]. Many studies thus limit their analysis to one gender only (e.g. [26]). Others in turn report that 2D:4D predicts the analyzed behavioral outcomes in men and not women or viceversa (e.g. [27]). The interplay of gender and experimental altruism is controversial: evidence exists that women give more than men, but this effect does not seem to be particularly robust (see [28] for an extensive review). Other papers note that women are more sensitive to the price of altruism [29] and are more expected to be fair [30]. In fact, Croson and Gneezy [28] conclude that women are more “*inequality averse*” and that “*women's decisions are more context-specific*” (p. 458). With these considerations in mind, we carefully analyze gender differences in the analysis below.

As for altruistic behavior, Millet and Dewitte [31] find both negative and positive relationships between giving and 2D:4D, depending on the mood they induce in their subjects, but they do not compare their results to any neutral control treatment and do not incentivize their subjects. Buser [32] finds positive correlation between 2D:4D and giving in DG, but he uses a self-reported index of 2D:4D and binary version of DG. This generates an imprecise measure of 2D:4D and precludes from exploiting nonlinearities. Other studies analyze the effects of 2D:4D on strategic behavior in Ultimatum, Public Good and/or Trust Games [32]–[36]. The ratio is also negatively related to certain types of asocial behavior such as aggression and some disorders associated with lower socialization such as autism, verbal fluency and depression (see [25] for a review), suggesting negative association between altruism and 2D:4D. Nevertheless, the differing conclusions across studies emphasize the extreme importance of sampling entire distributions, sufficiently large sample sizes and robustness analysis of reported findings.

In light of the above evidence, we conjecture that 2D:4D may be helpful in predicting individual altruism. In particular, due to above contradictory evidence we suspect that the association between fetal exposure and willingness to give might not be linear but non-monotonic. Moreover, we conjecture that this association will be gender-specific.

## Methods

### General Information

A total of 193 first-year undergraduate students participated in at least one of our experimental sessions during one academic year. The subjects were first-year undergraduate students (freshmen) of Economics at the University of Granada, Spain. The study was approved by the Ethical Committee of the Universidad de Granada and all subjects provided informed written consent (IC). The IC explains the content of the experiment they will perform and the payoffs attached to their performance. Anonymity was also assured and the Spanish law regarding data protection briefly explained.

The DGs were run twice with the same group of undergraduate students: (i) in the first week of their first academic year (before they get to know their classmates) in October 2010 and (ii) at the end of the academic year (after developing social relationships and after potentially learning from the first DG) in May 2011. Henceforth, we label each session 2010 and 2011, respectively. In both 2010 and 2011, all the four sections of the first year were visited and students were invited to participate in an economic experiment involving money. The participation was voluntary. Any individual who did not want to participate was allowed to leave the class before each session. Those willing to participate were seated separately, each with enough space to preserve anonymity, and they were provided with written instructions. We followed procedures similar to Brañas-Garza et al. [37]. First, we elicit their within-class social ties (without providing any incentives) and consequently invited them to play the DG. Each subject played the DG as the Dictator, dividing 5€ between herself and another randomly chosen individual from the list of all the participants of the experiment (independently of the attended section). Subjects were informed that each participant would potentially be either a Dictator or Recipient (but not both of them) with one half probability. Giving was expressed in real money up to two decimals.

After the experiment, subjects were invited one by one to an office for the payment and the scanning of their both hands. Both hands were scanned with a high-resolution scanner (Canon Slide 90). To determine 2D:4D, we measured the lengths of the index and ring digits on both hands from basal crease to the finger tip. To ensure the most accurate measurement, we measured the ratio from the scanned pictures twice. The first measurement was made right after the scanning, while the second was performed 14 months later, in January 2012. The data reported in this study use the average of both measures. The correlation between the average and the first (second) measure on the right hand is 0.97 (0.97) (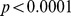 in both cases). The figures are 0.93 and 0.93, resp. (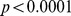) for left hands. As a robustness check, all the analysis was repeated using each measure separately and the results were unaffected.

We completed a sample of 173 and 148 participants in 2010 and 2011, respectively; 129 subjects participated in both sessions. Some subjects were excluded from the below analysis though. First, to ensure ethnical homogeneity, three non-Caucasian subjects were excluded from our data set. One of them only participated in 2010, one only in 2011, while the third participated in both. Their inclusion into the data set does not affect any of our results. Second, we do not include other 19 Caucasian subjects who participated in 2011 but not in 2010. They had no previous experience with the game and their behavior would not thus be comparable to the “experienced” subjects. Indeed, these 19 non-experienced Caucasian participants give on average 1.59€ more than other Caucasian participants in 2011 (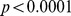). Third, since one male subject had his left-hand index finger broken in the past, we exclude him from the left-hand analysis. In sum, the analysis of right hands accounts for 171 subjects in 2010 (76 females) and 127 subjects in 2011 (58 females), whereas the left-hand data contain one male subject less. Women represent 44.44% of the sample 2010. 139 (out of 171) subjects reported their age; the average and median age in 2010 were 18.97 and 18 years, respectively (st.dev. 3.79; range between 18 and 60). The composition is similar in 2011.

Each participant was assigned a random identification number prior to the scanning and received a plastic card with an ID number. They were advised to keep it as their identification in future experiments and it served as an ID to record the experimental data and the digit ratios. In May 2011, we again visited the four classes and repeated the same experimental procedure (except the hand scanning). The data on altruism and digit ratios are available upon request from the authors.

The above data were combined with other characteristics of subjects collected in additional sessions. In April 2011, we ran the risk aversion session via an incentivized Holt and Laury's [38] protocol and at the beginning of June we invited the subjects to fill a questionnaire eliciting other characteristics, such as time preferences, socio-economic status etc., used as controls in the present study (see *Econometric Approach*).

In *Discussion*, we combine our results with a larger sample of digit ratios elicited one year later to be able to complement the analysis with a representative distribution of digit ratios in the population. The procedure of elicitation was identical as described above and we account for 440 males and 577 females in the sample. See the next section for details.

### Econometric Approach

To provide a rigorous statistical analysis of the experimental results, we perform a series of estimations. The dependent variables are all based on Dictators' giving in any of our sessions. Since there is evidence that people take from others in DGs if it is allowed [39] and giving is restricted to be non-negative in our experiment, our dependent variable is truncated from below by zero and we use censored regression analysis. All reported estimations were also reproduced using simple linear regression and using a logarithmic transformation of the dependent variable. The results are very similar and thus not reported here.

In particular, three types of models are estimated according to the dependent variable:

1. *Dictators' giving in 2010* and *2011*: continuous dependent variable (2 decimal places) censored from below by 0, cross-section, censored regression analysis, Tables 1 and 2.
2. *Dictators' giving in both 2010 and 2011*: continuous dependent variable (2 decimal places) censored from below by 0, cross-section and two periods, censored random-effect panel-data analysis, Table 3.
3. *The change of behavior from 2010 to 2011*, calculated as Dictators' giving in 2010 minus Dictators' giving in 2011: continuous dependent variable (2 decimal places) censored from below by −5 and above by 5, censored regression analysis (no censored observation in the data), Table 4.

**Table 1:** Dictator giving and digit ratio (2010), censored regression.

Right-hand digit ratio								
	(a)	(b)	(c)	(d)	(e)	(f)	(male)	(female)
2D:4D	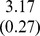	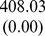	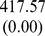	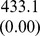	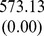	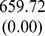	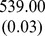	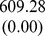
2D:4D^2^		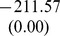	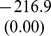	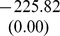	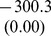	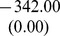	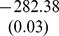	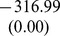
Female			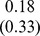	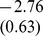	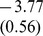	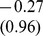		
Fem.*2D:4D				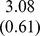	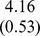	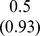		
Risk Aver.					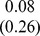	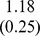	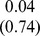	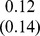
Other Heterogen.	No	No	No	No	No	Yes	No	No
N	171	171	171	171	149	107	88	61
Pseudo-R^2^	0.002	0.023	0.024	0.025	0.036	0.065	0.019	0.087
p (model)	0.263	0.003	0.006	0.012	0.006	0.000	0.113	0.003
**Left-hand digit ratio**								
	**(a)**	**(b)**	**(c)**	**(d)**	**(e)**	**(f)**	**(male)**	**(female)**
2D:4D	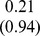	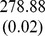	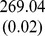	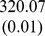	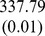	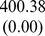	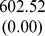	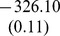
2D:4D^2^		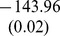	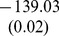	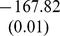	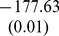	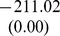	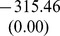	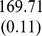
Female			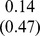	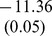	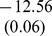	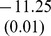		
Fem.*2D:4D				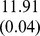	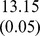	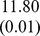		
Risk Aver.					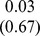	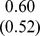	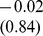	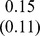
Other Heterogen.	No	No	No	No	No	Yes	No	No
N	170	170	170	170	148	106	87	61
Pseudo-R^2^	0	0.012	0.012	0.020	0.023	0.044	0.046	0.033
p (model)	0.939	0.056	0.098	0.034	0.063	-	0.005	0.137

p-values in parentheses. Constants non-reported: non-significant in (top a) (p = 0.58), in (bottom a) and (bottom f) (p>0.12), significant otherwise.

**Table 2:** Dictator giving and digit ratio (2011), censored regression.

Right-hand digit ratio								
	(a)	(b)	(c)	(d)	(e)	(f)	(male)	(female)
2D:4D	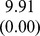	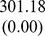	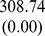	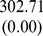	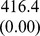	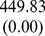	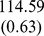	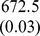
2D:4D^2^		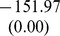	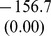	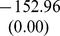	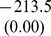	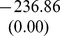	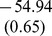	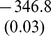
Female			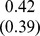	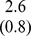	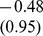	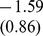		
Fem.*2D:4D				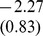	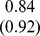	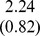		
Risk Aver.					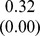	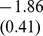	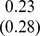	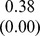
Other Heterogen.	No	No	No	No	No	Yes	No	No
N	127	127	127	127	122	107	69	53
Pseudo-R^2^	0.013	0.018	0.024	0.024	0.054	0.091	0.023	0.116
p (model)	0.000	0.000	0.000	0.000	0.000	0.000	0.000	0.000
**Left-hand digit ratio**								
	**(a)**	**(b)**	**(c)**	**(d)**	**(e)**	**(f)**	**(male)**	**(female)**
2D:4D	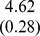	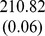	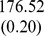	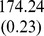	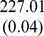	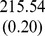	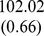	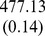
2D:4D^2^		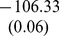	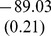	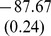	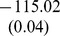	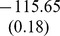	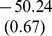	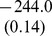
Female			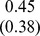	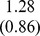	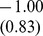	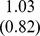		
Fem.*2D:4D				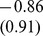	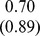	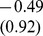		
Risk Aver.					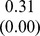	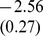	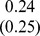	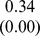
Other Heterogen.	No	No	No	No	No	Yes	No	No
N	126	126	126	126	121	106	68	53
Pseudo-R^2^	0.003	0.006	0.067	0.013	0.040	0.075	0.016	0.077
p (model)	0.281	0.121	0.000	–	–	–	0.030	0.000

p-values in parentheses. Constants non-significant in (top male)(p = 0.6), (bottom a), (bottom c–d),

(bottom f), (bottom male), (female) (p>0.19), significant otherwise.

**Table 3:** Dictator giving and 2D:4D, panel-data random-effects censored regression.

Right-hand digit ratio								
	(a)	(b)	(c)	(d)	(e)	(f)	(male)	(female)
2D:4D	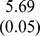	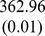	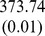	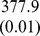	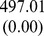	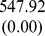	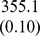	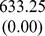
2D:4D^2^		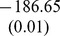	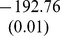	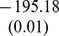	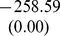	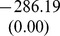	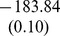	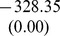
Female			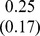	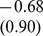	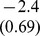	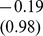		
Fem.*2D:4D				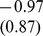	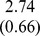	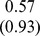		
Risk Aver.					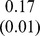	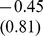	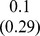	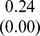
Other Heterogen.	No	No	No	No	No	Yes	No	No
N	171	171	171	171	149	107	88	61
p (model)	0.042	0.002	0.003	0.007	0.001	0.025	0.208	0.000
**Left-hand digit ratio**								
	**(a)**	**(b)**	**(c)**	**(d)**	**(e)**	**(f)**	**(male)**	**(female)**
2D:4D	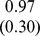	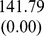	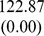	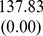	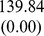	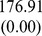	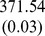	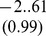
2D:4D^2^		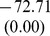	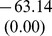	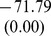	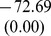	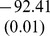	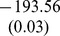	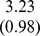
Female			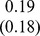	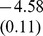	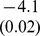	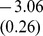		
Fem.*2D:4D				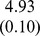	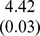	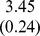		
Risk Aver.					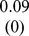	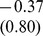	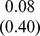	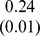
Other Heterogen.	No	No	No	No	No	Yes	No	No
N	170	170	170	170	148	106	87	61
p (model)	0.3	0.001	0.001	0	-	-	0.117	0.039

p-values in parentheses. Constants non-reported: non-significant in (bottom a, top/bottom male) (p>0.18), significant otherwise.

**Table 4:** The change of behavior (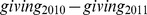) and digit ratio, censored regressions.

Right-hand digit ratio								
	(a)	(b)	(c)	(d)	(e)	(f)	(male)	(female)
2D:4D	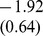	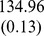	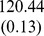	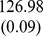	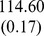	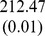	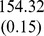	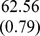
2D:4D^2^		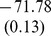	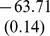	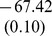	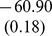	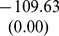	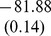	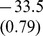
Female			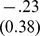	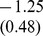	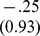	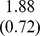		
Fem. *2D:4D				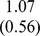	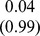	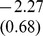		
Risk Aver.					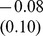	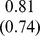	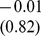	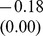
Other Heterogen.	No	No	No	No	No	Yes	No	No
N	125	125	125	125	120	105	67	53
Pseudo-R^2^	0.001	0.004	0.008	0.008	0.013	0.040	0.003	0.031
p (model)	0.636	0.274	0.142	-	-	-	0.246	0.000
**Left-hand digit ratio**								
	**(a)**	**(b)**	**(c)**	**(d)**	**(e)**	**(f)**	**(male)**	**(female)**
2D:4D	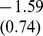	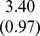	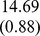	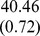	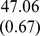	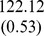	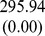	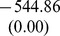
2D:4D^2^		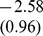	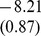	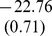	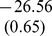	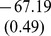	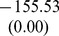	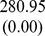
Female			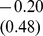	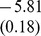	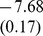	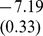		
Fem. *2D:4D				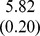	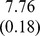	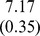		
Risk Aver.					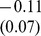	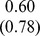	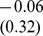	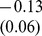
								
Other Heterogen.	No	No	No	No	No	Yes	No	No
N	125	125	125	125	120	105	67	53
Pseudo-R^2^	0.001	0.001	0.003	0.005	0.013	0.037	0.018	0.078
p (model)	0.744	0.947	0.889	-	-	-	0.000	0.000

p-values in parentheses. Constants non-reported: significant at 2% in all models except (top a) (p = 0.09), p>0.58 in (bottom a–f); p<0.0001 in (bottom male/female).

Each model is reported under eight different specifications: six models with the complete data set, (a)–(f), one model for the subsample of men, and one for women, (male) and (female). The structure of the independent variables is the same in the four specifications. The regressions are mainly focused on the role of 2D:4D, 2D:4D^2^, gender and risk aversion [27], [40]–[43]. In estimations (f), we also control for other variables that have been documented to influence either the 2D:4D and/or giving in the DG: intelligence [27], academic performance [44], time preferences [45], position in the class network [37], [46] and socioeconomic status.

As mentioned above, we combine our data with a different data set (see *Discussion*), where each gender-specific median, 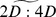, is used as a proxy for the population median. These medians are 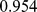 for males' and 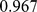 for females' right hands; the corresponding left-hand counterparts are 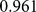 and 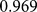, respectively. We used these numbers as proxies for the median 2D:4D in the population and relate giving in the DG to the deviation, in absolute terms, of individual 2D:4D from gender-specific population median 2D:4D's. The deviation variable in the estimated models in Table 5 is 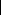 *2D:4D*–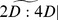 and (*2D:4D–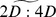
*)^2^is the deviation squared. There are three types of models depending on the way the deviation variable enter the regression and whether controls are included or not: (i) linear term alone (*a–b*) (ii) both linear and quadratic terms (*c–d*), (c) quadratic term alone (*e–f*). We also report the best estimations separated for men and women. The other regressors coincide with Tables 1–4.

**Table 5:** Dictator giving and the deviation from the population median of the 2D:4D censored regressions.

Right-hand digit ratio								
	(a)	(b)	(c)	(d)	(e)	(f)	(male)	(female)
2D:4D-2 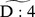 D	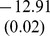	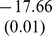	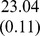	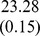				
(2D:4D-2 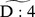 D)^2^			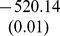	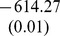	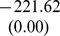	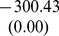	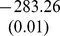	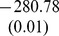
Female		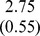		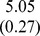		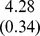		
Fem. *2D:4D		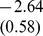		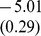		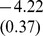		
Risk Aver.		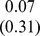		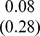		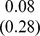	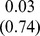	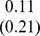
N	171	149	171	149	171	149	88	61
Pseudo-R^2^	0.013	0.025	0.026	0.040	0.022	0.036	0.020	0.078
p (model)	0.012	0.012	0.002	0.002	0.001	0.001	0.044	0.004
**Left-hand digit ratio**								
	**(a)**	**(b)**	**(c)**	**(d)**	**(e)**	**(f)**	**(male)**	**(female)**
2D:4D-2 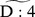 D	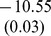	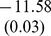	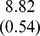	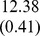				
(2D:4D-2 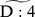 D)^2^			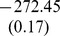	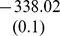	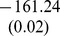	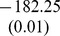	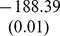	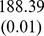
Female		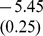		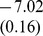		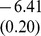		
Fem. *2D:4D		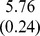		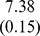		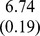		
Risk Aver.		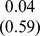		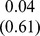		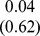	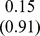	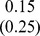
N	170	148	170	148	170	148	87	61
Pseudo-R^2^	0.010	0.017	0.014	0.023	0.014	0.021	0.043	0.031
p (model)	0.029	0.12	0.044	0.068	0.015	0.050	0.003	0.036

p-values in parentheses. Constants at 1% in all models except (top d) (p = 0.028) and (bottom female) (p = 0.25).

In all regressions, we report *p*-values based on estimated robust standard errors corrected for possible correlations within students from the same sections, as these individuals may have been under the influence of common factors and are more likely to know each other. In case of 2010 results (Table 1), the standard errors are robust but assumed uncorrelated (as people did not have time to know each other), but controlling for possible intra-section correlations has no effect on the regressions.

## Results

### Dictators' Giving

**Figure 1:**
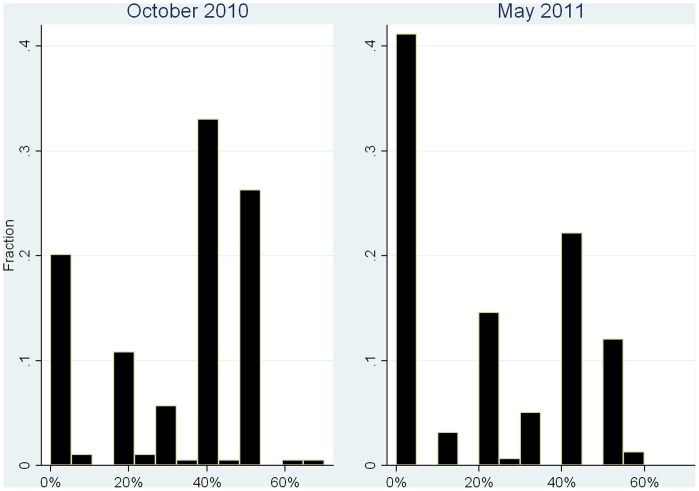
Dictator Giving in October 2010 and May 2011. *Left*: 2010. Mean: 1.62, St.Dev.: 0.99, Median: 2, 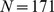 (76 females). *Right*: 2011. Mean: 0.89, St.Dev.: 0.97, Median: 1, 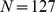 (58 females).

Figure 1 summarizes Dictators' giving in the experiment in 2010 (left, 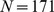) and 2011 (right, 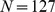). In 2010, the average Dictators' giving is 32.4% out of 5€, while they give on average 17.8% in 2011. Subjects are more selfish in 2011 than in 2010 (Wilcoxon signed-rank test: 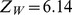, 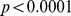; any other test leads to the same conclusion). On average, people gave 0.71€ (44.2%) less in 2011 than in 2010.

The pairwise correlation between the behavior of subjects who participated in both games is 0.327 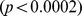, positive but far from 1. These differences may suggest that any relation found in one of the periods should disappear in the other one. As we shall see below, this is not the case.

Concerning gender, we observe no effect in 2010 (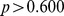 using 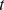
- and Wilcoxon unpaired rank-sum tests), but there seems to be marginal gender effects in 2011 (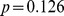, and 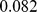 for the same tests, respectively). Men gave 0.79€ less (50.4%), while women passed 0.61€ less to the Recipients (37.1%). Women change the behavior slightly less, but this difference is not statistically significant (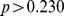 for any test).

### Digit Ratios

Males exhibit lower right-hand digit ratios (*Male*–Mean: 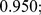 St.Dev.: 0.031; *Female*–Mean: 0.966; St.Dev.: 0.033). This gender effect is supported by any statistical test (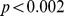 for the 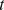
- and Wilcoxon unpaired rank-sum tests). The average 2D:4D's are 0.950 and 0.965 for men and women if we only consider participants in both Dictator Games. This difference is again significant (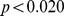). The reported distributions are statistically indistinguishable from [27] for men, women and the pooled data. This makes us confident that the observed population constitutes a representative sample.

As for the left hands, the gender effects are weaker but in the same direction. Males have lower 2D:4D's if we consider the whole sample at 6% (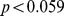 for the same tests; *Male*–Mean: 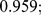 St.Dev.: 0.036; *Female* - Mean: 0.969; St.Dev.: 0.031). However, they are not significant for the participants in both DG sessions (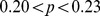). The averages are 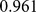 for males and 0.969 for females.

The correlations between the left and right 2D:4D are 0.657 in 2010 (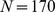) and 0.661 (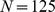); highly significant (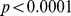) but far from one. These correlations are the same for males and females separately up to two decimals. Hence, the asymmetry does not seem to be gender-specific.

### Regression Analysis

**Figure 2:**
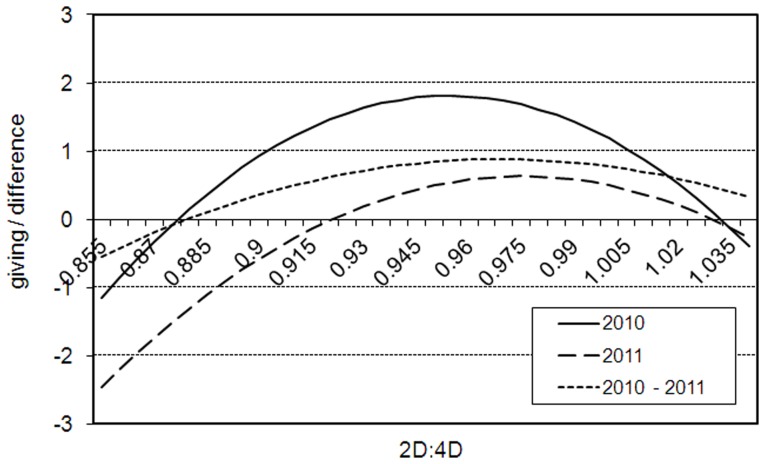
The estimated relation. Dictator's giving in 2010 (solid), 2011 (dashed) and updating of behavior from 2010 to 2011 (dotted) on 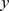-axis and 2D:4D (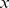-axis); censored regression analysis; right hands. Estimated results: (i) 2010: 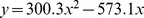, (ii) 2011: 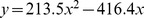, (iii) 2010–2011: 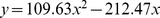. The reported estimations control for heterogeneity; control variables are held at their averages.

Tables 1–3 show the estimation results of the 2010, 2011 and the aggregated data for both right (top) and left hands (bottom), while Table 4 provides results for the change of behavior from 2010 to 2011. Figure 2 summarizes the right-hand results associating 2D:4D with giving in the two DGs and the change of behavior.

The linear relationship is positive but non-significant in 2010 for both hands (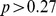), but once we introduce the squared 2D:4D the estimates reveal a non-monotonic, concave association between giving and 2D:4D: the most generous subjects have intermediate 2D:4D. These results remain for both left and right hands, if we estimate the models separately for men and women, and are robust to inclusion of controls that have shown to be related to altruism and/or the digit ratio in other studies. The unique exception is the model for female left hands where we find no significant association. In sum, the results are fairly robust to different specifications, different subsamples, and left/right hands. Since weaker left-hand effects are commonly observed in the literature, it serves as an indication of the robustness of our findings.

Since the behavior in DGs is generally sensitive to many details [14], we further test these findings. We repeated the experiment in 2011 with the same subject pool and the findings are qualitatively similar (Table 2), even though the subjects are significantly more selfish (see Figure 1). The differences we find are: (i) the linear relationship between giving and right-hand 2D:4D becomes significant in 2011 (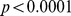); (ii) the association gives up being non-monotonic for male right-hand 2D:4D and turns out to be linear (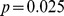; pseudo-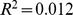; model 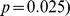; and (iii) the left-hand estimates are statistically weaker in 2011 than in 2010.

The conclusions are reinforced if we treat the data as a panel as shown in Table 3. Hence, there is a robust non-monotonic association between altruism and 2D:4D in our data.

Another interesting result is associated to learning; that is, how subjects update their behavior. As illustrated in Table 4, the 2D:4D also exerts non-monotonic influence on the change of behavior from 2010 to 2011 if we control for individual heterogeneity. The linear relation is never significant, but adding the squared 2D:4D results in lower 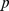-values of the linear term. In case of right hands, the linear and quadratic terms are jointly significant at 10% in Model (d) and at 1% in Model (f), in which we control for individual heterogeneity more systematically. Subjects with intermediate right-hand 2D:4D, i.e. the most generous subjects, tend to adjust their giving downwards more that individuals with low and high 2D:4D's. These results have to be enjoyed with care though as 2D:4D does not exert influence on giving in several of our model specifications.

Note that the relation is gender-specific in case of left hands. The association remains inverted U-shaped for men, but for women we find a highly significant U-shaped (rather than inverted U-shaped) relation. This explains why we never observe significant effects in the pooled estimations. As the dependent variable is not statistically different across genders and women exhibit inverted U-shaped association using right hands, we suspect that this result has to do with the difference between left and right hands. However, since it is not well understood how fetal hormones manifest through left vs. right hands, we cannot interpret this finding.

One may argue that an inverted U-shaped association can potentially be an artifact of low sharing of subjects with high and low 2D:4D's in 2010 who simply might not be allowed to give any less in 2011 given the design. Nevertheless, such an explanation can be contrasted with the U-shaped association observed using female left hands, even though female left-hand 2D:4D does not seem to predict giving in the DG.

In addition, note that there are only 125 observations in Table 4. We removed two male subjects with extremely much higher giving in 2010 than in 2011, as their inclusion dramatically improves the estimates. Nevertheless, since these results are highly sensitive to the removal of these two outliers, we report the conservative and more robust estimates in Table 4, which are robust to further removals.

Males receive more prenatal testosterone and less oestrogen than females, reflected in lower 2D:4D's in men [25]. Hence, the relation between 2D:4D and giving might potentially explain gender effects observed in Dictator Games [28]. Regressing Dictator giving in 2011 only on female dummy (and the constant term) never leads to statistically significant effects of gender on giving in our data (regressions not reported). Thus, the influence of 2D:4D on giving behavior is orthogonal to these gender effects documented elsewhere and scholars cannot capture the detected biological predisposition by controlling for gender.

## Discussion

We provide support for the hypothesis that 2D:4D may predict altruistic behavior. This is implied by the non-monotonic association we find between 2D:4D and giving in Dictator game. In contrast to the 2D:4D literature that reports important differences between men and women and between right and left hands, our findings are for the most part robust to these issues.

Our results corroborate the idea that part of the variation of human altruism is already determined by prenatal events. This sugests that biological and genetic factors play an important role in social norm transmission (as much as cultural transmission and socialization). Our results are in line with the analysis of Benjamin et al. [8] who conclude that the genetic variation in behavioral traits will most likely be explained by many factors with each having a small effect. The McFadden's pseudo-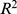 from the 2010 estimations suggest that 2D:4D alone explains 2.3% of the individual variation in giving, while gender improves the fit by 0.2% and controlling for heterogeneity more systematically leads to final 6.5%. The absolute numbers should be treated with caution and interpreted relatively, due to the general difficulties of interpreting the pseudo-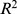 [47]. For comparison, 2D:4D has relatively similar effects in ordinary least-squares estimations of the same models. The 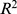
's are 0.059 (compared to 0.023 in the censored regressions), 0.064 (compared to 0.025 while controlling for gender) and 0.1658 (compared to 0.065 while controlling for heterogeneity more systematically).

Note that our analysis differs from other studies relating prosocial behavior and biological factors such as circulating hormones [48] or oxytocin [49]. Their levels are endogenous, complicating causality assessments. That is why we chose to work with the exposure to prenatal sex hormones, since they are not systematically related to their circulating counterparts [50].

We would like to emphasize that the degree of exposure to prenatal sex hormones and thus 2D:4D ratio, as much as any other biological traits in humans and non-humans [10]–[12], has most likely been tuned by thousands years of evolution till it has reached an “optimal” level. Does the distance from the mean predict a subjects' adherence to a desirable sharing norm?

We address this question in the following manner. We combine our data with a large distribution of digit ratios of individuals from another study. This is gives us a total of 1017 observations (577 females) (see *Methods*). The right-hand 2D:4D's that maximize giving in 2010 (before subjects learn and may know the other participants) are 0.956 and 0.961 for men and women, respectively (see Figure 2). These figures are very close to 0.957 and 0.969, the proxies for the median 2D:4D's in the population.

We further provide a more rigorous test. We estimate the relation between giving and (the absolute value of) the deviation from the above population medians. The linear term is significant on its own in Table 5. However, the best model in terms of model significance, adjusted-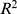, and 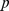-values associated to 2D:4D variables (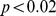) turns out to be regressing giving over the quadratic term for both hands. Controlling for heterogeneity in this model reinforces this conclusion. With one exception, we observe a decreasing concave association, suggesting that the higher the distance from the optimal value the lower the giving, but at a decreasing rate. Hence, the distance from the median 2D:4D relates negatively to the observed sharing behavior. We find the contrary - increasing convex association - for deviations of female left-hand 2D:4D's from the population median.

One possible interpretation of the above findings comes from stabilizing selection. Since sharing with others is socially beneficial, selfish individuals are socially excluded and their fitness affected negatively. If individuals who are exposed too much or too little do not share with others, there is an evolutionary pressure on these non-altruistic individuals, which in turn generates an indirect evolutionary pressures on the degree of exposure to prenatal sex hormones by raising survival probabilities of individuals with intermediate levels of exposure. This hypothesis is supported by observed distributions of 2D:4D in the literature, which are universally concentrated around the median values [25].

Even though the previous paragraphs provide certain support for our hypothesis, a word of caution is in place here. First, our results are rather suggestive. They only provide one piece of evidence to support such argument and cannot be taken as conclusive evidence of stabilizing selection. Other explanations are obviously possible. Second, we know that exposure to fetal testosterone and oestrogen conditions many behavioral and physical traits in humans (not only sharing behavior). The 2D:4D optimal from the evolutionary perspective (if it exists) could thus be confounded with effects on these traits and potential trade-offs have to be taken into account. Therefore, we have to be wary of making general conclusions based on our exercise. On the other hand, some studies have already suggested non-monotonic impacts of 2D:4D on some behavioral outcomes (e.g. [13], [40], [51], [52]).
